# Mapping of Small Nerve Trunks and Branches Using Adaptive Flexible Electrodes

**DOI:** 10.1002/advs.201500386

**Published:** 2016-03-23

**Authors:** Zhuolin Xiang, Swathi Sheshadri, Sang‐Hoon Lee, Jiahui Wang, Ning Xue, Nitish V. Thakor, Shih‐Cheng Yen, Chengkuo Lee

**Affiliations:** ^1^ Department of Electrical and Computer EngineeringNational University of Singapore4 Engineering Drive 3Singapore117583Singapore; ^2^ Singapore Institute for Neurotechnology (SiNAPSE)National University of Singapore28 Medical Drive, #05‐CORSingapore117456Singapore; ^3^ Center for Intelligent Sensors and MEMSNational University of Singapore4 Engineering Drive 3Singapore117576Singapore; ^4^ Institute of Microelectronics (IME)Agency for Science, Technology and Research (A*STAR)11 Science Park Road, Singapore Science Park IISingapore117685Singapore; ^5^ Department of Biomedical EngineeringSchool of MedicineJohns Hopkins University BaltimoreMD21205USA

**Keywords:** electroceuticals, flexible electronics, nerve mapping, peripheral neural interface, small diameter nerves

## Abstract

**Selective stimulation is delivered to the sciatic nerve** using different paris of contacts on a split‐ring electrode, while simulatneous recordings are acquired by the neural ribbon electrodes on three different branches. Two hook electrodes are also implanted in the muscle to monitor the activated muscle responses. It shows that the high precision implantation of electrodes, increases the efficacy and reduces the incidence of side effects.

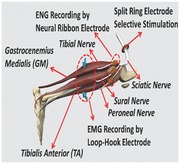

Flexible biomedical devices currently become popular since they are promising in direct contact with delicate soft tissues and biological structures,^1^ such as wearable triboelectrical nanogenerators,^2^, ^3^, ^4^ epidermal electrical sensors,^5^, ^6^, ^7^ and implanted electrical stimulators.^8^, ^9^, ^10^ Functions provided by these flexible devices, including energy harvesting, physiological signal sensing, and nanomedicine delivery, help researchers to explore new approaches to further understand disease mechanisms and develop new therapies. This new trend is known as electroceuticals.^11^ One of the most popular example is using electrical impulses to interface with the nervous system to modulate organ function as a form of treatment for certain diseases,^12^, ^13^, ^14^, ^15^ including hypertension,^16^, ^17^ uremia,^18^ diabetes,^19^ and Parkinson disease.^20^ Some examples include targeting the greater splanchnic nerve branches innervating the adrenal medulla to treat diabetes,^21^, ^22^ the pelvic nerve innervating the urinary bladder to treat bladder dysfunction,^23^, ^24^ and the carotid sinus nerve branches to treat hypoxia.^25^, ^26^

These nerves are typically made up of axons from hundreds of nanometers to several micrometers.^27^, ^28^ Even the entire nerves are very small (50–300 μm in diameter), and often not easily accessible.^29^, ^30^ This makes it challenging to implant electronic devices chronically on these nerves, especially if wireless power and wireless data telemetry (which have typically effective distances of less than 20 mm^31^) are used. One strategy is then to interface with larger nerve trunks like the vagus, splanchnic, or pelvic nerves, which are much more accessible. However, when stimulating the nerve trunks, it is extremely critical that appropriate nerve fibers in the nerve trunk are stimulated, while unrelated nerve fibers within the nerve trunk are not stimulated. The best way to evaluate the stimulation effects will be to directly measure organ output, e.g., plasma content,^32^ urine,^33^ catecholamines,^34^ norepinephrine spillover,^35^ etc., to make sure that organ functions controlled by those unrelated nerve fibers are not being modulated inappropriately. However, these measures are often not immediately available (for instance, stimulation of the adrenal medulla is reflected in increased levels of catecholamines after 10–60 min), and may thus require extended time to reposition the stimulation device, or worse, require additional surgeries to reposition the stimulating device on the nerve trunk if it turns out that the initial position was suboptimal.

An alternative would be to electrically map the nerve trunk by performing selective stimulation of the nerve trunk and simultaneously recording evoked signals from the nerve branches. This allows the position of the stimulating electrodes to be optimized quickly by observing the effect of the stimulation on the relevant nerve branches. The electrodes on the nerve branches can then be removed so that only the electrodes on the nerve trunk are chronically implanted. However, this has been challenging to perform as most peripheral neural interfaces, such as penetrating electrodes,^36^, ^37^, ^38^, ^39^ regenerative electrodes,^40^, ^41^, ^42^ and extra‐neural electrodes^43^, ^44^ were designed to interface with relatively large nerves (with diameters larger than 500 μm). For example, penetrating electrodes such as intrafascicular electrodes typically require the use of a needle to penetrate the nerve, but these needles are too bulky to pass through the very small nerve branches. In the most commonly used extraneural electrodes, cuff electrodes, the stiffness of the platinum wires and polydimethylsiloxane sleeves prevents the electrode from fully conforming to these small nerves, thus leading to poor signal quality.

In this study, we will demonstrate how an innovative neural ribbon (NR) electrode can be used to record from small nerves with different diameters so that a relatively large nerve trunk can be mapped. Unlike traditional neural interfaces like the cuff electrode that may produce compression of the nerve, this innovative neural ribbon electrode can be wrapped around the nerve, with only sutures holding down the two ends. Its unique, spiral wrapping mechanism allows the same device to record from nerves with different diameters. Since the whole device is extremely flexible, and because the electrical traces are fabricated using a thin film metal layer rather than wires, it can be easily handled during the implantation, and still be fully conformal to nerve branches that are less than 300 μm.

Since the sciatic nerve is the largest and most accessible peripheral nerve trunk in the rat, we chose it to test our concept for mapping the nerve trunk by recording from the nerve branches. In addition, unlike the vagus or splanchnic nerves, which innervate organs and have effects like increases in catecholamine release that are not easy to measure, the sciatic nerve innervates muscles, and we can easily verify the nerve branch recordings by recording the downstream muscle contractions evoked by the nerve stimulation. The implantation locations of the neural ribbon (**Figure** **1**b) and a split‐ring electrode^45^ (SR, Figure 1c) are shown in **Figure** **2**. Eight contacts on the neural ribbon electrode were used to record nerve signals (electroneurogram, ENG) from the following nerve branches: peroneal nerve (≈400 μm in diameter), tibial nerve (≈600 μm in diameter), and sural nerve (≈250 μm in diameter). Meanwhile, the split‐ring electrode, which had six independent electrode contacts, was implanted on the sciatic nerve. By stimulating different pairs of electrode contacts on the split‐ring electrode, different current paths could be formed in the sciatic nerve. These different current paths were able to activate different portions of the sciatic nerve and deliver selective stimulation to different groups of fascicles in the sciatic nerve. Two hook electrodes were also implanted into the belly of the gastrocenemius medialis muscle (GM) and tibialis anterior muscle (TA) to record electromyography (EMG) signals. Since the GM is innervated by tibial nerve, and the TA is innervated by the peroneal nerve, the EMG signals recorded by the implanted hook electrodes were used to verify whether the ENG signals from the nerve branches matched the EMG signals from the associated muscles.

**Figure 1:**
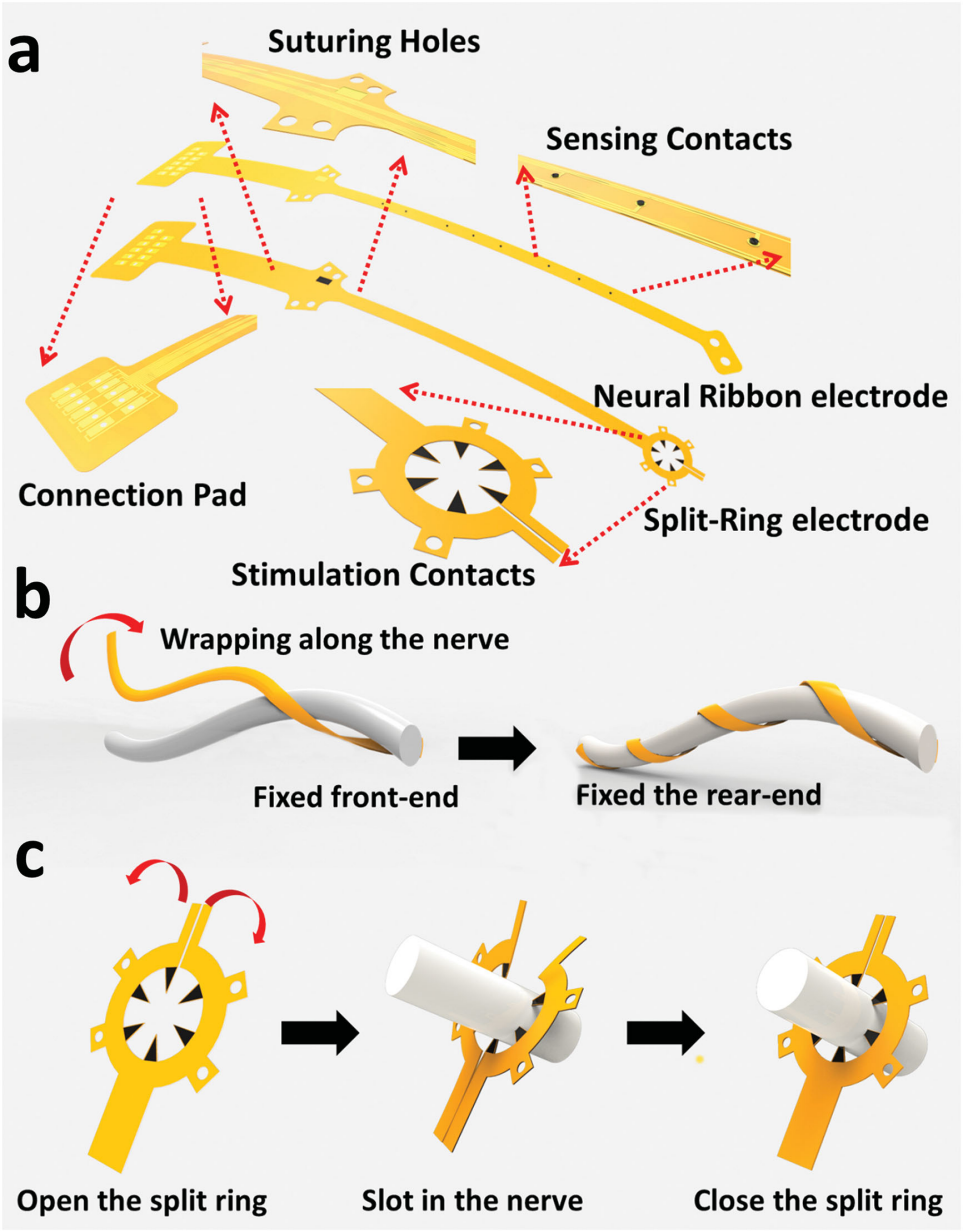
Illustration of the neural ribbon electrode and the split‐ring electrode. a) Both the neural ribbon electrode and the split‐ring electrode used in the experiments were strip‐like devices. Suture holes were designed to fix the device on the nerve. A transition portion on the electrode was intentionally added between the connection pad and the rear suture holes to reduce the interference from the connector during implantation. b) Front suture holes were used to fix the front part of the neural ribbon to the nerve. With this part fixed, the neural ribbon was wrapped along the nerve helically. As a result, the recording contacts came into direct contact with the epineurium surface, maximizing its ability to record neural signals. c) After the split‐ring electrode was opened by slightly bending its fringe in opposite directions, the nerve was slotted inside. The ring fringe then holds the nerve, and the six protruding contacts came into direct contact with the epineurium.

**Figure 2:**
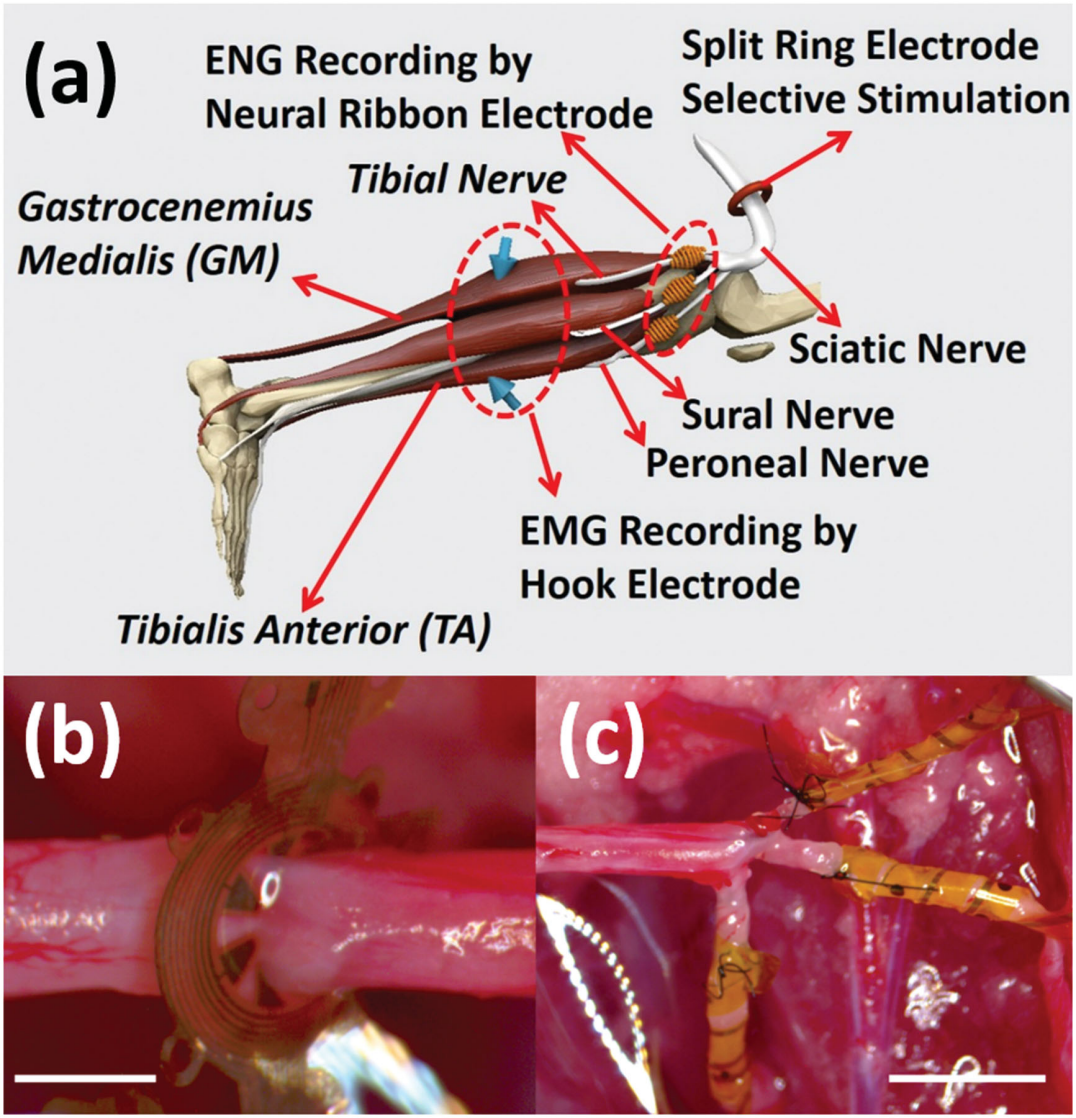
Implantation of the neural ribbon electrode and split‐ring electrode. a) Schematic drawing of the implantation location of the neural ribbon electrodes, split‐ring electrode, and hook electrodes. The split‐ring electrode was attached to the sciatic nerve to perform selective stimulation of the sciatic nerve. Three neural ribbon electrodes were implanted on three nerve branches to record ENG signals: the peroneal nerve, the tibial nerve, and the sural nerve. Hook electrodes were implanted in the gastrocenemius medialis (GM) and tibialis anterior (TA) muscles to record EMG signals. b) Image of the implantation of the split‐ring electrode (Scale bar: 1 mm). c) Image of the implantation of the neural ribbon electrodes (Scale bar: 3 mm).

We first delivered 20 μs cathodic monophasic pulses of varying current amplitudes (0.2–1 mA) to the main sciatic nerve using the split‐ring electrode. **Figure** **3** shows the representative data when SR Contacts 1 and 2 on the split‐ring electrode were used as the anode and cathode, respectively, with a stimulation current of 0.6 mA. Figure 3a,b shows ENG signals recorded from NR Contact 3 on the neural ribbon electrode implanted on the peroneal nerve (the recordings from tibial nerve and sural nerve have similar profile but different amplitudes), and EMG signals recorded from the hook electrode implanted in the TA muscle, respectively. 60 evoked compound action potentials (CAPs) from the nerve and muscle were recorded and averaged together to reduce noise in the recordings. The stimulation was delivered at time 0, and the corresponding stimulus artifact appeared almost immediately on both the channels of the neural ribbon electrode and the hook electrode. The averaged evoked CAPs for the eight different electrode contacts on the neural ribbon electrode implanted on the peroneal nerve are shown in **Figure** **4**c. The ENG signals recorded on the different channels varied in duration and amplitude probably for a variety of reasons. First, since the neural ribbon wrapped around the nerve, the eight electrode contacts touched different positions on the nerve. Evoked ENG signals travelled along the nerve and were recorded by these contacts at different latencies. In addition, the fascicles activated by the stimulation were likely anisotropically distributed under the epineurium, thus causing the distance (and as a result, the amplitudes of the signals) between the sensing electrode contacts and the active fascicles to vary at each of the electrode contacts. In Figure 3d, plots were constructed from the averaged EMGs recorded simultaneously from the GM and TA muscles. Due to the location of the implantation, we found the amplitude of the EMG from the GM muscle to be considerably larger than those from the TA muscles. Additional EMG and ENG recordings corresponding to different stimulation amplitudes on different pairs of contacts on the split‐ring electrodes are shown in the Supporting Information.

**Figure 3:**
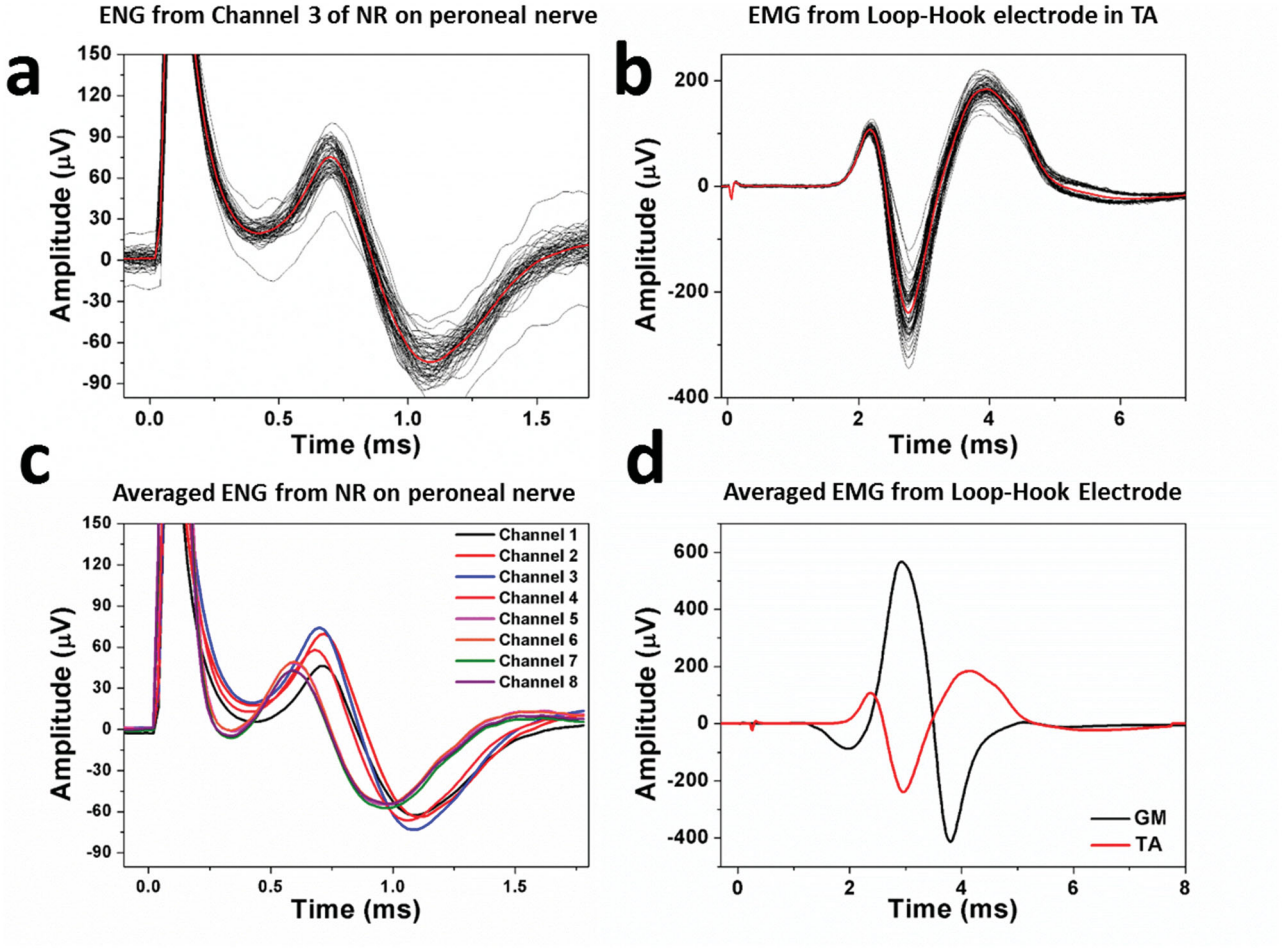
Representative neural and muscle recordings. a) 60 evoked ENG signals recorded by the neural ribbon electrode on the peroneal nerve and the averaged data (plotted in red). b) 60 evoked EMG signals recorded by the hook electrode in the TA muscle, and the averaged data (plotted in red). c) ENG signals recorded from eight channels on the same neural ribbon electrode. d) EMG signals recorded from the two hook electrodes.

**Figure 4:**
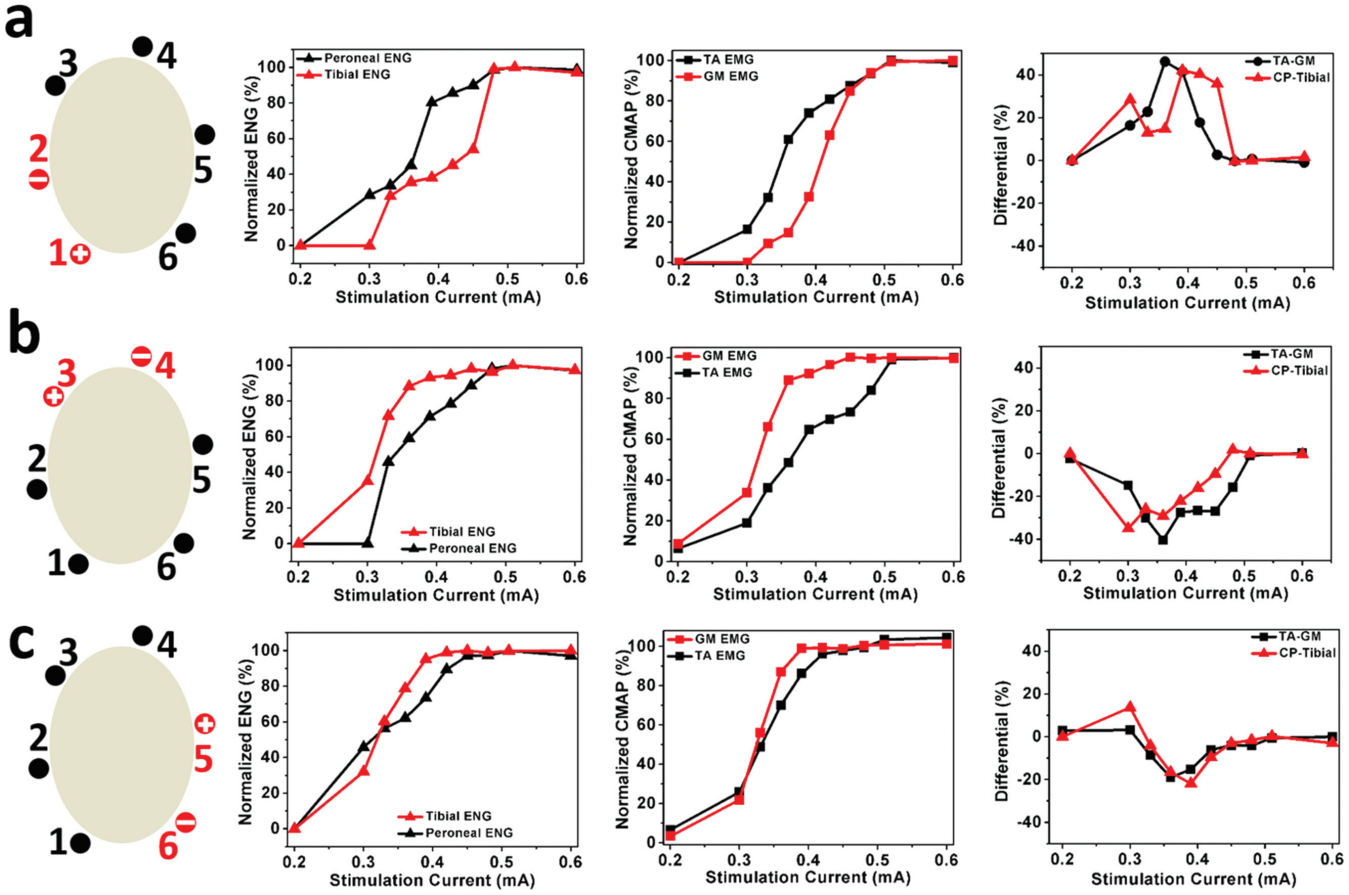
Recorded ENG and EMG signals when stimulating different sets of contacts. When stimulating a) Contact 1 and Contact 2, b) Contact 3 and Contact 4, and c) Contact 5 and Contact 6, the plots (from left to right) show signals from the peroneal nerve (CP) and the tibial nerve, normalized EMG signals in the TA and GM muscles, and the difference in the ENG signals from the peroneal nerve and tibial nerve, as well as the difference in activation of the TA and GM muscles.

After demonstrating that we were able to stimulate the sciatic nerve and record ENGs and EMGs from the neural ribbon and hook electrodes, we proceeded to map the organization of the sciatic nerve. Figure 4 shows the differences in the ENG and EMG recordings when we stimulated different pairs of contacts on the split‐ring electrode. For instance, in Figure 4a, we used SR Contacts 1 and 2 (highlighted in red) as the anode and cathode, respectively. The plot on the left shows the ENGs recorded in the peroneal and tibial nerves as we increased the stimulation current. As there were eight electrode contacts each on the neural ribbon electrode implanted on the peroneal and tibial nerves, we chose the contact that gave us the largest ENG signal (e.g., in Figure 4c, that would be NR Contact 3). In this case, it is clear that when stimulating SR Contacts 1 and 2 with low current amplitudes, the nerve fibers activated first in the sciatic nerves were those that branched into the peroneal nerve. As the current amplitudes increased, more fibers in the sciatic nerve that branched into the peroneal nerve were activated, which led to larger evoked amplitudes being recorded in the peroneal nerve. However, at the same time, it appeared that some of the fibers that branched into the tibial nerve were also activated, leading to reduced specificity. At even larger current amplitudes, it appeared that the activation of the fibers that branched into the peroneal nerve had saturated, and increasing the stimulation amplitudes only served to activate more of the fibers that branched into the tibial nerve. An analogous scenario can be observed in the EMG recordings shown in the plot in the middle in Figure 4a. Activation of the fibers leading to the peroneal nerve led to activation of the TA muscle. However, as the current amplitudes increased, the GM muscle became more and more activated due to activation of the fibers leading to the tibial nerve.

We characterized the ability of the stimulation in the sciatic nerve to differentially target fibers leading to the peroneal nerve as opposed to those leading to the tibial nerve by computing a measure that characterized the difference in the amplitudes of the ENG in the peroneal and tibial nerves. This is shown in the red line in the plot on the right in Figure 4a. As the stimulation current increased on SR Contacts 1 and 2 on the split‐ring electrode, the difference between the activation of the peroneal and tibial nerve increased to a maximum of 40% between current amplitudes of 0.39 and 0.45 mA (please see the Supporting Information for more details). Further increases in the stimulation amplitudes resulted in poor differentiation. These results clearly show that if maximal peroneal activation with minimal tibial activation was desired, stimulating SR Contacts 1 and 2 with amplitudes around 0.4 mA would be best. Similar results using the EMG signals are shown in the black line on the same plot.

Figure 4b shows the results when SR Contacts 3 and 4 were stimulated, while Figure 4c shows the results for SR Contacts 5 and 6. In both these cases, stimulation of the sciatic nerve using these contacts evoked more tibial nerve activation than peroneal nerve activation, with optimal stimulation currents around 0.3–0.36 mA and 0.36–0.39 mA, respectively (please see the Supporting Information for more details).

To obtain a better understanding of the different stimulation configurations on the sciatic nerve trunk and their effects on the peroneal, tibial, and sural nerve branches, we computed a selectivity index, SI*_i_*, to quantify the activation of one nerve branch, S*_i_*, relative to the sum of the activation of all three nerve branches

(1) $$SI_{i}=\frac{S_{i}}{\sum S_{j}}$$

This measure peaks at a value of 1 when only the nerve branch of interest is activated, drops down to a value of 0.33 when all three nerve branches are activated equally, and can be as low as 0 if the nerve branch of interest is not activated at all. **Figure** **5**a–c shows the selectivity index in the peroneal nerve, tibial nerve, and sural nerve, respectively, with different stimulation currents and configurations. For instance, in Figure 5a, it shows that the strongest activation of the peroneal nerve, with minimal activation of the tibial and sural nerves, was achieved when SR Contacts 1 and 2 were stimulated with a current of 0.3 mA. Similarly, Figure 5b shows that the strongest activation of the tibial nerve, with minimal activation of the peroneal and sural nerves, was achieved when SR Contacts 3 and 4 were stimulated with a current of 0.3 mA. For the sural nerve, **Figure** **6**c shows that the selectivity was a lot weaker than the other two, with selectivity indices close to 0.3. This means that activation of the sural nerve was usually accompanied by activation of the peroneal and tibial nerves. The highest selectivity found for the sural nerve was when SR Contacts 1 and 4 were stimulated with a current of 0.63–0.69 mA.

**Figure 5:**
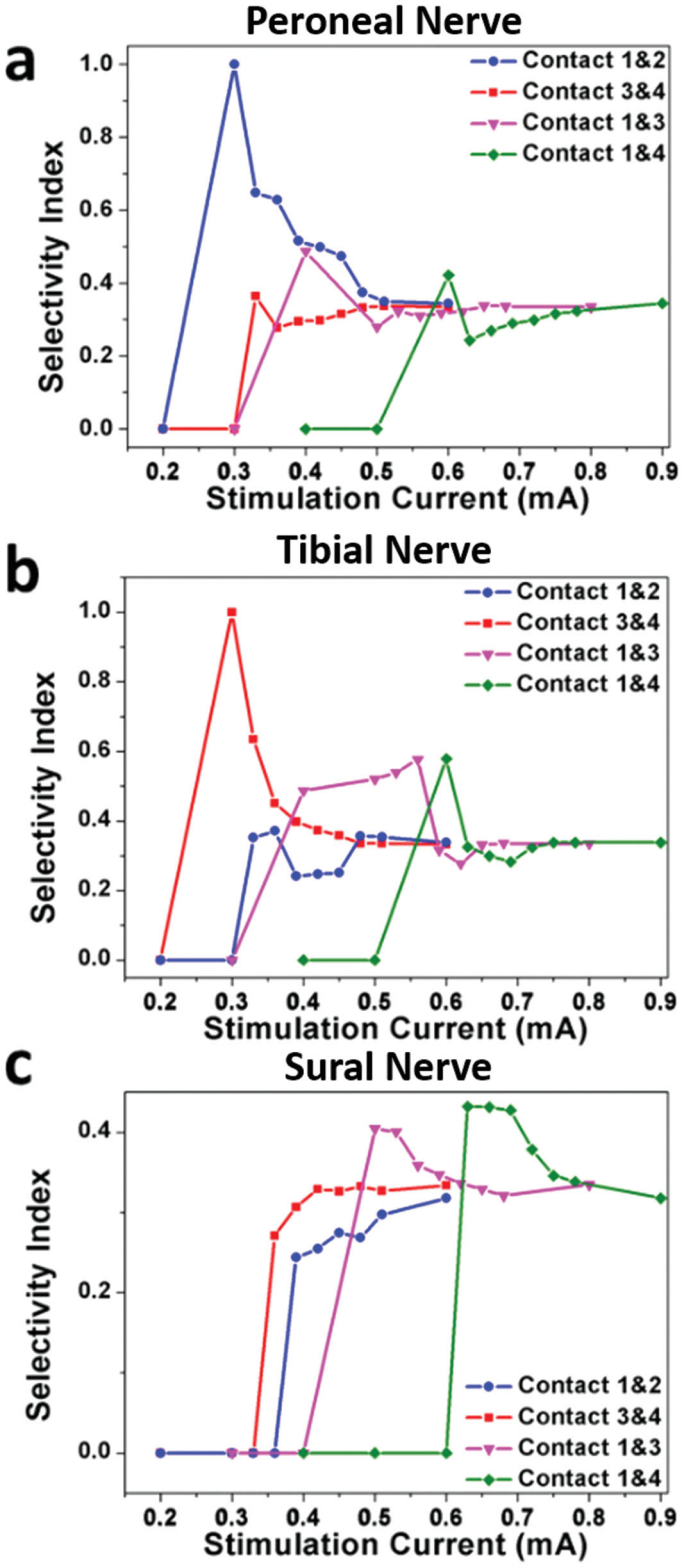
Selectivity indices under different stimulation currents and different stimulation configurations. Selective index for a) peroneal nerve, b) tibial nerve, and (c) sural nerve.

**Figure 6:**
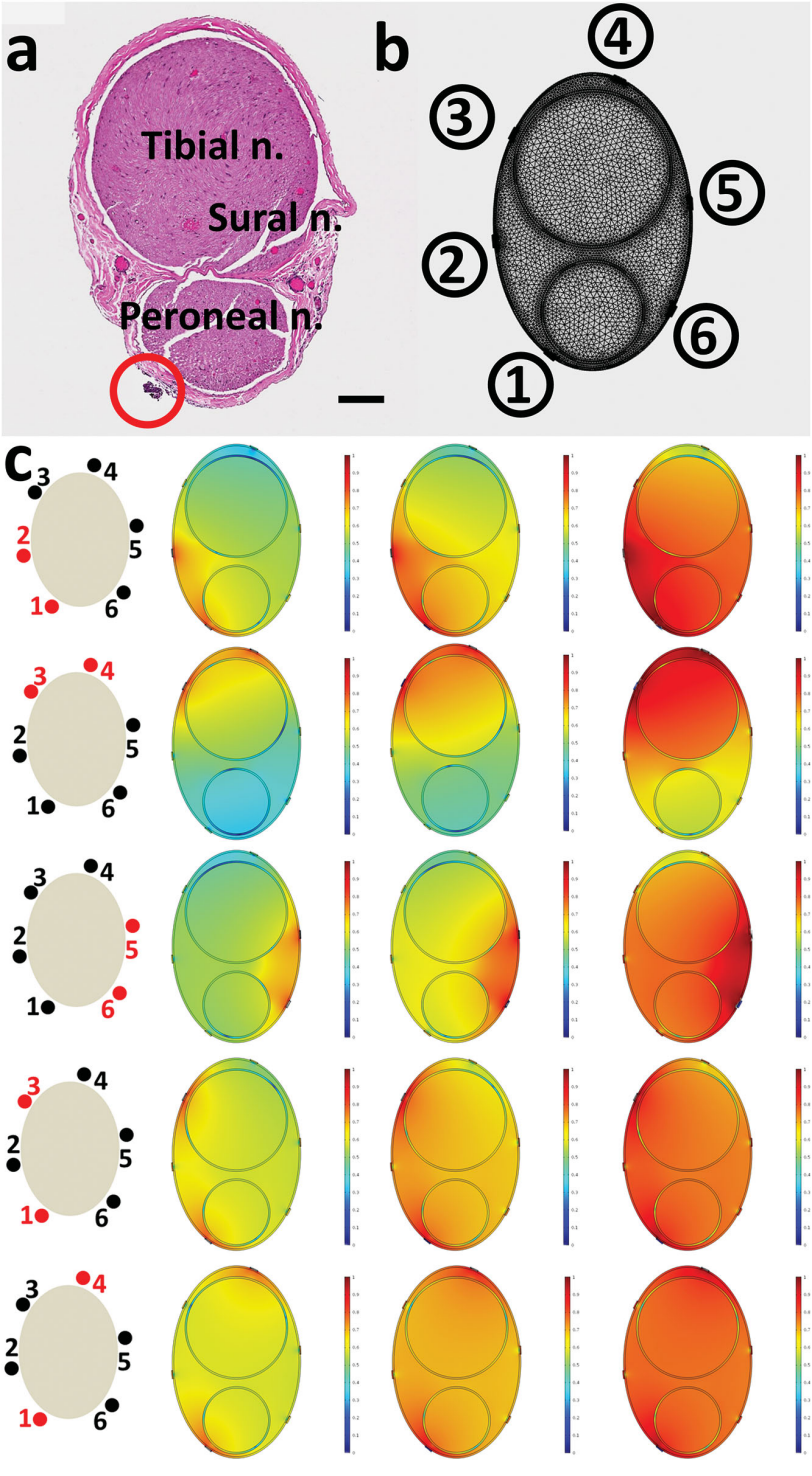
Histology and model of the nerve trunk. a) Histology image for the sciatic nerve used in the experiments above (Scale bar: 100 μm). b) Mesh model of the implanted split‐ring electrode and the nerve. c) Current density distribution in the simulated nerve with the same geometry and dimension. Under each stimulation contact combination, images from left to right are simulation results with 0.3, 0.5, and 0.8 mA stimulation.

We studied the geometry of the sciatic nerve to gain a better understanding of our stimulation results. After a small lesion was made on the sciatic nerve trunk with SR Contact 1 to mark the position of the split‐ring electrode on the sciatic nerve, the sciatic nerve was transected, fixed, and stained with hematoxylin and eosin dyes to obtain the histology image shown in Figure 6a. The position of SR Contact 1 is highlighted by the red circle. Using the topographical pattern defined by Badia et al.^46^ for the rat sciatic nerve, we labeled the fascicles in the sciatic nerve that branched into the peroneal nerve, tibial nerve, and sural nerve branches. A finite element method (FEM) model was used to calculate the current density generated by stimulating with different pairs of contacts on the split‐ring electrode. As shown in Figure 6b, a 3D model with the same geometry as that shown in the histology image was constructed in Comsol Multiphysics 5. In the simulation, the electrical properties of geometrical domains were based on published values (refer to the Supporting Information).^47^ The perineurium was modeled as a distributed resistance boundary with a thickness equal to 3% of the fascicle diameter.^48^ The simulated current density distribution is shown in Figure 6c. For each combination of contacts, the amplitude of the stimulation current increased from left to right. As the current increased, the spread of current inside the nerve increased, resulting in more nerve fascicles being activated. This is consistent with our results that showed that with large current amplitudes, fascicles were activated indiscriminately, resulting in low selectivity indices (Figure 5). The simulations also showed that when two nearby contacts on the split‐ring electrode were stimulated, the stimulation current density was more concentrated and localized. This concentrated current distribution resulted in nerve fascicles being more easily activated, which is consistent with the lower stimulation thresholds we found (Figures 3, 4, 5). The simulations also showed that SR Contacts 1 and 2 were closer to the peroneal nerve fascicles while SR Contacts 3 and 4 were closer to the tibial nerve fascicles. This supports the results we found in Figure 5a that SR Contacts 1 and 2 led to the highest selectivity in the peroneal nerve, and in Figure 5b, that SR Contacts 3 and 4 led to the highest selectivity in the tibial nerve. The high degree of agreement between the histology image and the FEM results provided us with confidence that the recordings from the neural ribbon electrodes provided an accurate mapping of the sciatic nerve.

In this study, we have shown that we are able to stimulate a nerve trunk selectively and record evoked neural activation signals in several nerve branches simultaneously. This was made possible using a highly novel neural ribbon electrode that, for the first time, allows us to record from small nerve branches as small as 250 μm. We have shown that with neural ribbon electrodes implanted simultaneously on multiple nerve branches, we were able to identify the optimal stimulation configurations and currents on the nerve trunk to selectively activate any one out of a number of nerve branches, while simultaneously reducing (or avoiding) the stimulation of the other off‐target nerve branches. The results were confirmed with both end‐target activation (in this case, muscle EMG), as well as histological analysis.

We believe these results demonstrate that our setup may greatly simplify the challenge of mapping a nerve trunk for the purposes of positioning a chronic neuromodulatory device. By temporarily affixing the neural ribbon electrodes simultaneously on both on‐target nerve branches and off‐target nerve branches, the optimal position, stimulation configuration, and stimulation parameters on the nerve trunk to maximize activation of the on‐target nerve branch and minimize activation of the off‐target nerve branches can be quickly determined. This will be a much simpler and more immediate alternative to measuring protein levels in blood or other physiological measurements.

Although we have only used the neural ribbon electrode for recording, these electrodes can also be used for electrical stimulation. Compared to the split‐ring electrode, which needs to be customized to the diameter of the nerve, the same neural ribbon electrode can be used with nerves of different sizes. In addition, the angular separation between electrode sites on the nerve can be easily controlled by altering the angle at which the electrode wraps around the nerve.

In our experiments, we found that we were able to achieve the highest selectivity with low amplitude current stimulation on the nerve trunk using pairs of neighboring electrodes, while larger amplitude currents recruited too many nerve fibers and caused decreases in selectivity. The selectivity may be further increased by increasing the number of contacts (and thus decreasing the separation between neighboring electrodes) on the stimulating electrode, which should allow more localized stimulation of the nerve to occur when pairs of neighboring electrodes are used.

There are certainly a great many cases in which on‐target and off‐target effects are not so easily delineated into on‐target nerve branches and off‐target nerve branches. In those cases, our solution may be of limited utility. However, with the recent surge in interest in electroceuticals, we believe our solution will be just one of the many techniques that can be deployed, and it will be incredibly exciting for us to see how other innovative clinicians and scientists apply our solution.

## Experimental Section

*Design of the Neural Ribbon and Split‐Ring Electrode*: The flexible neural ribbon electrode is illustrated in Figure 1a. The strip‐like device had two suture holes on the front end of the ribbon, and four suture holes on the rear end. These suture holes were used to fix the device to the epineurium surface. There were eight electrical sensing contacts on the ribbon between the front and rear suture holes. The electrical sensing contacts were 150 μm in diameter, with 1.5 mm spacing between them. A 0.5 cm transition area beyond the rear suture holes was designed to minimize the interference from the connector during the implantation process. A connection pad with through holes was designed to match with a customized Omnetics (Omnetics Connector Corp., MN, USA) connector. After the post contacts on the Omnetics connector were aligned with these holes and pushed through, a drop of silver paste and medical‐grade UV adhesive (Henkel, Germany) ensured a reliable electrical connection was made. Both ENG and EMG recordings were taken with respect to an Ag/AgCl wire sutured under the skin beside the surgical site.

For selective stimulation of the nerve trunk, a split‐ring electrode with six contacts was designed as shown in Figure 1a. The connection pad and the rear suture holes were similar to the neural ribbon electrode mentioned before. However, a 2D ring frame was designed to encircle the nerve at its front‐end. The inner diameter of the ring structure was slightly larger than the diameter of the target nerve. Six protruding probes from the inner edge of the ring frame touched the epineurium, and delivered electrical current into the nerve. By connecting different pairs of the six protruding probes to the current stimulator (DigiTimer, Digitimer Ltd, UK), stimulation current was introduced into different parts of the nerve to stimulate different fascicles.

During implantation, the neural ribbon electrode and the split‐ring electrode were attached to the nerve as shown in Figure 1b,c. After the front suture holes were used to fix the front part of the neural ribbon electrode onto the nerve, the device was then wrapped along the nerve body helically. This was possible due to the high flexibility of the ultrathin polyimide substrate^49^ (Figure 1b). At the same time, electrical sensing contacts on the neural ribbon electrode directly touched the epineurium surface, establishing excellent contact with the nerve. After the neural ribbon was fully wrapped onto a nerve, the rear suture holes were used to fix the rear part of the electrode onto the epineurium surface.

For the split‐ring electrode, there was a 20 μm gap on its ring frame, which enabled the split‐ring electrode to be opened by slightly bending the fringes in opposite directions (Figure 1c). Then a nerve was slotted inside the split‐ring electrode, with the hope that all six protruding probes made stable contact with the epineurium. After releasing the bending force that opened the split‐ring electrodes, the electrode closed up and was then fixed on the nerve. There were four suture holes around the ring frame, which could be used to attach the device to the nerve for chronic implantation. The fabrication of these two electrodes was based on standard microelectromechanical system processes and is discussed in the Supporting Information.

*Electrophysiology*: Sprague–Dawley rats (250–400 g) were used for the sciatic nerve implantations. Anesthesia was induced using a mixture of Xylazine (7.5 mg kg^−1^ intraperitoneal (IP)) and Ketamine (50 mg kg^−1^ IP) in 0.9% NaCl. After the animals were fully anesthetized, the legs were shaved from the knee to the hip by an electrical shaver. The surgical field was disinfected with chlorhexidine and 70% ethanol. Then, the femur of the rat was found, and an incision of ≈0.5 cm parallel and ≈1.5 mm anterior to the femur was made by a surgical blade. The underlying fat was removed, and the muscles close to the femur were separated with two autoclaved wooden sticks. When the embedded sciatic nerve was isolated, the attached fat was removed by surgical forceps. A 9‐0 suture with a curved needle was guided through one of the front suture holes. The curved needle penetrated the epineurium and took the suture into the nerve tissue. Then, the curved needle returned from the other front suture hole and tied a knot to fix this front part on the nerve. With this part fixed, the neural ribbon was wrapped along the nerve helically. After all the recording contacts touched the nerve surface, the rear suture holes were used to fix the other end of neural ribbon to the epineurium of the nerve. In addition, EMG hook electrodes (from Microprobes for Life Sciences Inc., Maryland, USA) were implanted in the gastrocenemius medialis (GM) and tibialis anterior (TA) muscles to record EMG signals. These electrodes were slightly modified to include a loop in the wire that allowed us to suture the electrode more securely to the muscle to prevent electrode detachment during muscle movement.

## Supporting information

As a service to our authors and readers, this journal provides supporting information supplied by the authors. Such materials are peer reviewed and may be re‐organized for online delivery, but are not copy‐edited or typeset. Technical support issues arising from supporting information (other than missing files) should be addressed to the authors.

Supplementary
