# Supplementary material for: Mapping of Small Nerve Trunks and Branches Using Adaptive Flexible Electrodes
Source: Adv Sci (Weinh). 2016 Mar 23;3(9):1500386. doi: 10.1002/advs.201500386 (PMC5039981; doi:10.1002/advs.201500386)
Supplement: Supplementary file 1 — Supplementary [file ADVS-3-0b-s001.pdf]

Copyright WILEY-VCH Verlag GmbH & Co. KGaA, 69469 Weinheim, Germany, 2016.

ADVANCED SCIENCE
Open Access

Supporting Information

for *Adv. Sci.*, DOI: 10.1002/advs.201500386

Mapping of Small Nerve Trunks and Branches Using Adaptive Flexible Electrodes

Zhuolin Xiang, Swathi Sheshadri, Sang-Hoon Lee, Jiahui Wang, Ning Xue, Nitish V. Thakor, Shih-Cheng Yen, and Chengkuo Lee**

Supporting Information

Mapping of Small Nerve Trunks and Branches Using Adaptive Flexible Electrodes

Zhuolin Xiang, Swathi Sheshadri, Sang-Hoon Lee, Jiahui Wang, Ning Xue, Nitish V. Thakor, Shih-Cheng Yen and Chengkuo Lee**

Zhuolin Xiang, Sang-Hoon Lee, Jiahui Wang and Prof. Chengkuo Lee
Department of Electrical & Computer Engineering, National University of Singapore, 4 Engineering Drive 3, Singapore 117583
Singapore Institute for Neurotechnology (SiNAPSE), National University of Singapore, 28 Medical Drive, #05-COR, Singapore 117456
Center for Intelligent Sensors and MEMS, National University of Singapore, 4 Engineering Drive 3, Singapore 117576
Email : elelc@nus.edu.sg

Swathi Sheshadri, Prof. Shih-Cheng Yen
Department of Electrical & Computer Engineering, National University of Singapore, 4 Engineering Drive 3, Singapore 117583
Singapore Institute for Neurotechnology (SiNAPSE), National University of Singapore, 28 Medical Drive, #05-COR, Singapore 117456
E-mail: shihcheng@nus.edu.sg

Dr. Ning Xue
Institute of Microelectronics (IME), Agency for Science, Technology and Research (A*STAR), 11 Science Park Road, Singapore Science Park II, Singapore 117685

Prof. Nitish V. Thakor
Department of Electrical & Computer Engineering, National University of Singapore, 4 Engineering Drive 3, Singapore 117583
Singapore Institute for Neurotechnology (SiNAPSE), National University of Singapore, 28 Medical Drive, #05-COR, Singapore 117456
Department of Biomedical Engineering, School of Medicine, Johns Hopkins University Baltimore, MD 21205, USA

Table 1. Raw data from ENG and EMG recordings

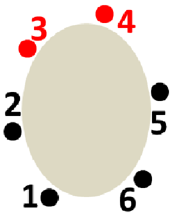

Stimulation	EMG		ENG		
I (mA)	TA(μV)	GM(μV)	Peroneal (μV)	Tibial (μV)	Sural (μV)
0.20	24.1±0.16	85.4±0.51	0	0	0
0.30	71.2±0.28	327.5±1.21	0	23.5±0.07	0
0.33	135.6±1.03	642.1±0.87	21.3±0.08	48.2±0.25	0
0.36	182.4±0.78	863.8±1.21	27.5±0.11	59.3±0.21	20.5±0.05
0.39	243.1±0.73	895.2±0.97	33.1±0.17	62.7±0.18	26.3±0.21
0.42	262.4±1.12	937.5±1.31	36.5±0.14	63.5±0.27	31.4±0.13
0.45	275.3±1.16	973.2±0.79	41.2±0.16	65.9±0.24	33.7±0.21
0.48	315.6±0.84	968.6±0.95	45.7±0.21	64.8±0.31	35.1±0.17
0.51	372.4±0.47	971.7±1.41	46.5±0.28	67.2±0.16	32.6±0.14
0.60	375.4±0.92	969.2±0.89	45.2±0.14	65.5±0.24	34.5±0.18

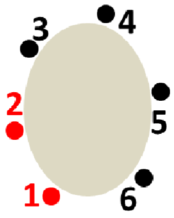

Stimulation	EMG		ENG		
I (mA)	TA(μV)	GM(μV)	Peroneal (μV)	Tibial (μV)	Sural (μV)
0.20	0	0	0	0	0
0.30	65.6±0.32	0	19.7±0.05	0	0
0.33	128.9±0.51	90.5±0.32	23.4±0.07	15.8±0.05	0
0.36	244.5±0.64	142.2±0.44	31.2±0.04	23.1±0.03	0
0.39	296.8±0.92	312.5±0.91	55.6±0.12	29.1±0.07	18.5±0.02
0.42	324.2±0.71	607.2±1.12	59.3±0.08	34.5±0.05	21.2±0.03
0.45	351.2±0.62	817.6±0.85	62.3±0.04	41.2±0.12	24.5±0.01
0.48	375.4±0.84	903.2±1.04	68.4±0.08	75.6±0.07	26.1±0.02
0.51	401.5±0.74	957.1±1.18	69.3±0.12	76.3±0.11	29.5±0.01
0.60	397.2±0.62	962.3±1.25	68.4±0.07	74.1±0.08	33.5±0.02

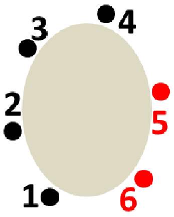

Stimulation	EMG		ENG		
I (mA)	TA(μV)	GM(μV)	Peroneal (μV)	Tibial (μV)	Sural (μV)
0.20	23.5±0.02	32.7±0.03	0	0	0
0.30	92.7±0.32	211.5±0.87	23.2±0.01	24.1±0.03	0
0.33	175.4±0.75	542.1±1.03	28.5±0.01	45.3±0.07	18.4±0.01
0.36	251.3±0.92	842.1±0.94	31.5±0.04	59.1±0.05	22.5±0.01
0.39	309.4±1.02	957.2±1.17	37.2±0.06	71.5±0.07	24.2±0.01
0.42	345.1±1.12	961.3±1.04	45.3±0.08	74.3±0.11	31.2±0.02
0.45	351.2±0.97	956.2±0.74	49.2±0.15	75.1±0.13	32.7±0.01
0.48	357.2±0.59	972.3±0.47	49.3±0.09	74.2±0.08	33.2±0.02
0.51	371.2±0.74	975.3±0.87	50.7±0.13	74.9±0.14	34.2±0.01
0.60	375.2±0.95	979.1±0.81	49.2±0.12	75.1±0.12	35.2±0.01

	Stimulation	EMG		ENG		
	I (mA)	TA(μV)	GM(μV)	Peroneal (μV)	Tibial (μV)	Sural (μV)
	0.30	25.2±0.02	0	0	0	0
	0.40	71.3±0.15	85.1±0.75	21.2±0.01	25.1±0.02	0
	0.50	92.5±0.09	174.2±0.62	23.1±0.01	31.2±0.01	18.2±0.01
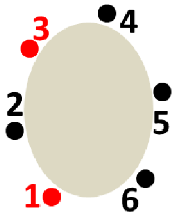	0.53	137.2±0.17	243.1±0.37	34.1±0.04	40.7±0.07	24.2±0.02
	0.56	231.5±0.21	536.2±0.95	43.2±0.08	61.4±0.16	27.3±0.01
	0.59	252.3±0.15	627.5±0.75	47.2±0.15	68.1±0.21	28.5±0.02
	0.62	332.5±0.54	915.2±1.13	52.1±0.21	75.3±0.15	29.2±0.01
	0.65	347.1±0.37	923.6±0.85	53.2±0.16	74.2±0.19	29.5±0.01
	0.68	372.6±0.74	931.2±1.09	54.7±0.13	77.2±0.25	30.2±0.02
	0.80	375.4±0.68	927.2±0.94	55.2±0.24	76.2±0.23	29.7±0.01
	Stimulation	EMG		ENG		
	I (mA)	TA(μV)	GM(μV)	Peroneal (μV)	Tibial (μV)	Sural (μV)
	0.40	0	0	0	0	0
	0.50	0	0	0	0	0
	0.60	51.2±0.42	235.2±0.65	18.2±0.02	25.1±0.01	0
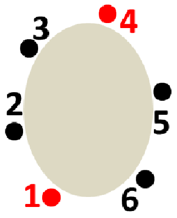	0.63	102.7±0.74	246.3±0.45	21.3±0.01	31.2±0.01	21.5±0.01
	0.66	152.3±0.65	342.1±0.62	25.9±0.01	34.9±0.02	24.9±0.02
	0.69	242.8±0.76	526.8±0.98	31.5±0.02	40.3±0.07	29.7±0.01
	0.72	273.2±0.81	665.1±1.08	38.6±0.04	55.2±0.09	31.2±0.02
	0.75	362.3±1.12	896.3±1.15	45.4±0.17	61.2±0.15	32.1±0.01
	0.78	374.2±1.05	927.8±1.23	50.8±0.24	65.2±0.17	31.4±0.02
	0.90	379.6±0.95	967.2±0.87	49.7±0.18	64.8±0.12	31.8±0.02

Table 2. Simulation Parameter

Component	Conductivity (S/m)
Perineurium	8.3×10^{-4}
Epineurium	8.2×10^{-1}
Fascicle	5.7×10^{-1}
Tissue	6.6×10^{-2}

Fabrication process for the neural ribbon electrode

The fabrication process follows standard photolithographic and clean room procedures. The detailed process is shown in Figure S1. Firstly, a 1 μm thick aluminum (Al) layer was evaporated onto the silicon substrate by physical vapor deposition (Figure S1(a)). It acted as a sacrificial layer to release the final device from the substrate. Then a 5 μm base layer of photosensitive polyimide (Durimide 7005, Fujifilm, Japan) was spun onto the Al coated substrate with a speed of 2000 rpm. After exposure under ultraviolet (UV) with a dosage of 120 mJ/cm^2, the base layer was post-baked and developed in HTRD2 and RER 600 (Fujifilm, Japan), which defined the bottom layer pattern of the neural ribbon (Figure S1(b)). The base polyimide layer was cured at 300 °C in N_2 for 0.5 h. This baking process was designed to only partially evaporate the water in the polyimide layer. In this way, it would create a chemically and physically stable surface for further processing, while still leaving some unterminated bonds to attach the top polyimide layer[1]. After that, a layer of AZ 9260 (AZ Electronic Materials, USA) was spun onto the polyimide base layer. This AZ layer was exposed and the electrode traces were patterned. A layer of 20 nm chrome (Cr) was deposited to improve the adhesion of the next conduction layer by sputtering. After a 350 nm thick gold layer was deposited (Figure S1(c)), the conductive metal layer was patterned by lift-off process in acetone (Figure S1(d)). Another 5 μm thick top layer of polyimide was spun onto the processed metal layer and patterned to expose the sensing contacts and connection pad (Figure S1(e)). The inset in Figure S1(e) demonstrates the concave openings of the contacts. Then, a 20 μm thick SU-8 2025 was spun onto the polyimide layer. After development, the SU-8 protruding bumps were patterned exactly on the top of the sensing contacts (Figure S1(g)). A stainless steel shadow mask was applied to the surface of the patterned neural ribbon device. Since the diameter of the holes on the shadow mask was larger than the diameter of the SU-8 bumps, the SU-8 bumps were fully exposed with careful alignment. Next, the chip covered with the shadow mask was loaded on a rotational and inclined stage in

the sputtering chamber. The SU-8 bumps, as well as the underlying sensing contacts, were sputtered with a layer of 300 nm Au (Figure S1(h)). In this way, the protruding SU-8 bumps were turned into conductive structures. The conventional approach to remove the sacrificial layer is through a wet etching process. However, the residue stress leads the released thin film structure to deform[2]. Instead, we adopted an anodic metal dissolution approach to release the whole device that not only could ensure a flat planar structure was released, but also was significantly faster than the traditional wet etching process[3]. The detailed release process has been described in our previous work[4]. Briefly, the wafer was immersed in a 2 M NaCl solution and connected to an external positive terminal of a voltage source at 1 V. A platinum (Pt) mesh electrode was connected to the negative terminal. A magnetic stir bar was also put inside the solution to keep the concentration of NaCl uniform. After around 20 minutes, the exposed portions of the Al sacrificial layer were removed, and only the covered portions of the Al sacrificial layer were left. Since the contact area between the Al sacrificial layer and the NaCl solution decreased, the current dropped, and the Al etching rate was reduced. Thus, the voltage was then increased to 20 V to speed up the release process. After the entire Al sacrificial layer was removed in 2 hours, the final device was released (Figure S1(i)). Then, the released device was packaged and a layer of carbon nanotubes were electroplated onto the electrode contacts to minimize the electrode impedance (Figure S1(j)).

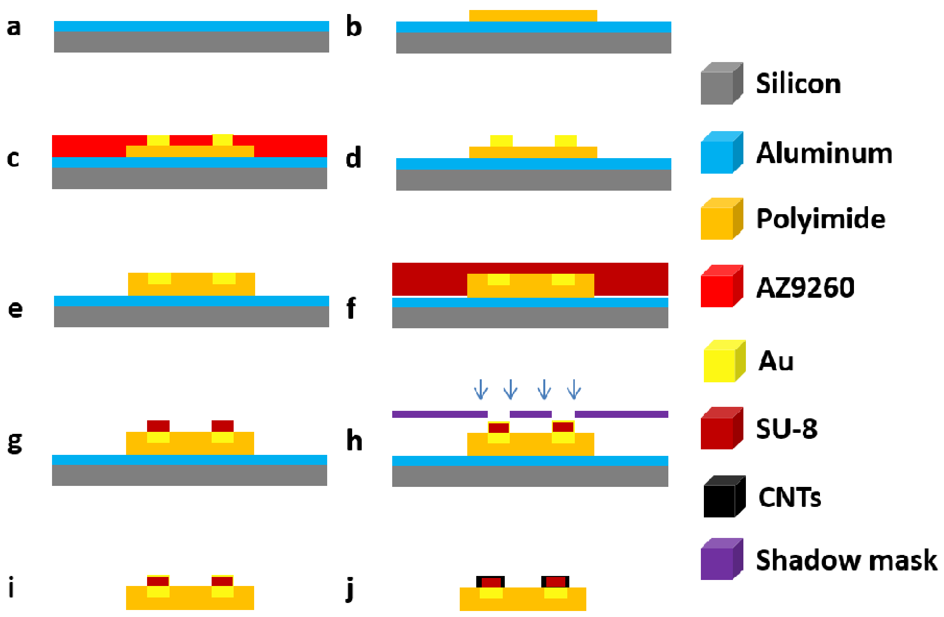

Figure S1. Fabrication process of the neural ribbon electrode.

Verification of neural signals

In order to verify that the signals recorded by the neural ribbon electrodes were neural signals, a nerve blocker (1% lidocaine) was applied to the nerve trunk distal to the split-ring electrode. After 10 minutes, all the neural signals on the nerve branches evoked by the nerve stimulation disappeared. One example of 0.6 mA stimulation through Contact 1 and Contact 2 is shown in Figure S2. It is a comparison of recorded signals from the tibial nerve before and after the nerve blocker was applied. Without the nerve blocker, the evoked ENG signals were obvious after the artifact (Figure S2 (a)). However, after the nerve blocker was applied, these neural signals vanished (Figure S2 (b)).

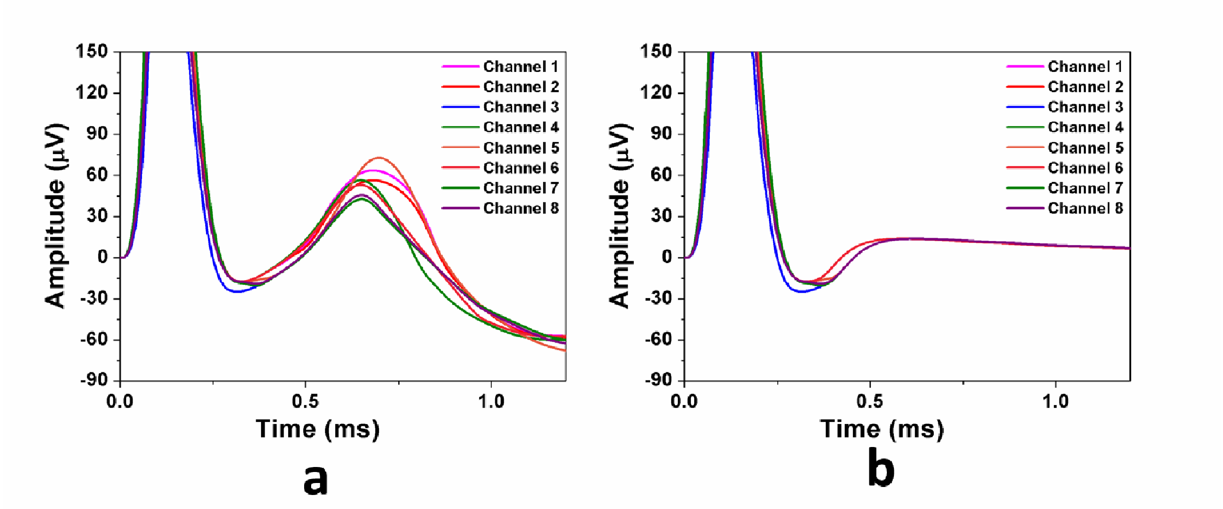

Figure S2 Comparison of neural activity before and after nerve blocker was applied.

Velocity measurement is another approach to verify that the recorded signal was neural in nature. During the experiment, the latency and the distance between the split-ring electrode and neural ribbon electrode were measured to calculate the nerve conduction velocity. The latency for the CAP signal in each implanted neural ribbon was obtained under different stimulation. The result is shown in Figure S3. Since the neural ribbon electrodes that were implanted on the nerve branches were 2.5 cm away from the split-ring electrode, the conduction velocity was computed to be around 35 m/s ~ 40 m/s, which agrees with the values reported by Stanley *et al*[5].

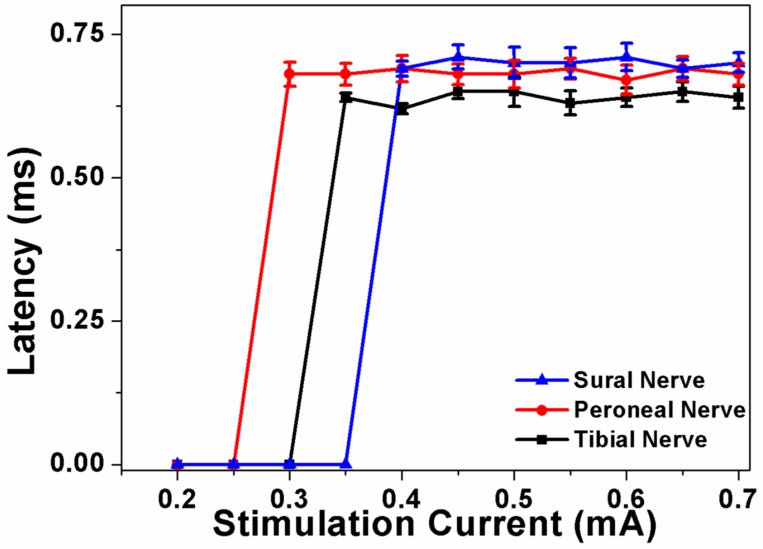

Figure S3 Latency measured on different nerve branches.

Electrical characterization

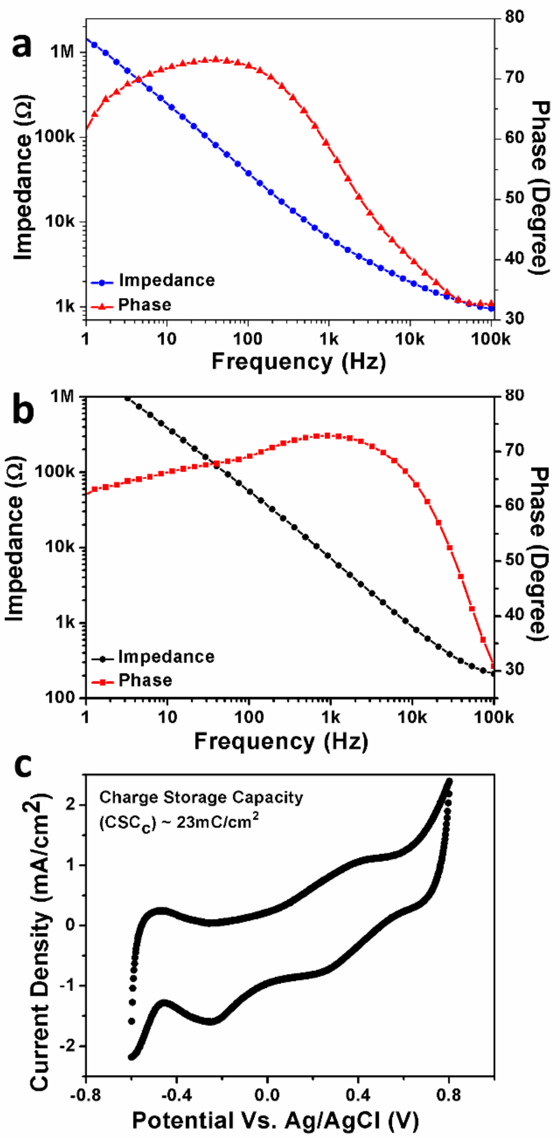

Figure S4. EIS and CV testing for the neural ribbon electrode and the split-ring electrode. (a)EIS testing for the neural ribbon electrode (b)(c) EIS and CV test for the split-ring electrode.

Electrochemical impedance spectroscopy (EIS) was conducted to investigate the electrical characteristics of the neural ribbon electrode, as well as the split-ring electrode. Phosphate buffered saline (PBS) was used as the medium. A sinusoidal wave with an amplitude of 50 mV and frequencies from 0.7 Hz to 100 kHz was applied. Three electrode configuration was adopted in the testing procedure with a sliver/silver chloride (Ag/AgCl) electrode acting as a reference, while a Platinum wire served as the counter electrode. The output impedance was recorded in vitro with Autolab PGSTAT100N and plotted here. At 1

kHz, the impedance for the neural ribbon electrode was 6.2 kΩ, while the impedance for the split-ring electrode was 7.1 kΩ. With the same testing setup, the charge storage capacity (CSC) of the split-ring electrode was evaluated by cyclic voltammetry (CV) test under a sweep rate of 50 mV/s. As shown in Figure S4(c), the CSC was calculated from the time integral of the cathodic current in CV over a potential range within the water electrolysis window. The CSC of the split-ring electrode was 23mC/cm^2.

[1] D. F. Lemmerhirt, E. M. Staudacher, K. D. Wise, *IEEE Trans. Biomed. Eng.* **2006**, *53*, 2084.

[2] W. Fang, J. A. Wickert, *J. Micromechanics Microengineering* **1996**, *6*, 301.

[3] S. Metz, A. Bertsch, P. Renaud, *J. Microelectromechanical Syst.* **2005**, *14*, 383.

[4] Z. Xiang, S.-C. Yen, N. Xue, T. Sun, W. M. Tsang, S. Zhang, L.-D. Liao, N. V Thakor, C. Lee, *J. Micromechanics Microengineering* **2014**, *24*, 065015.

[5] E. F. Stanley, *Exp. Neurol.* **1981**, *71*, 497.
